# Editorial: Chronic large-vessel occlusion in cerebrovascular disease: pathophysiology, diagnosis, and therapeutic advances

**DOI:** 10.3389/fneur.2026.2004605

**Published:** 2026-10-29

**Authors:** Weili Li, Jun Zhang

**Affiliations:** 1 Department of Neurology, Cerebrovascular Diseases Research Institute, Weifang People's Hospital, The First School of Clinical Medicine, Shandong Second Medical University, Weifang, China; 2 Department of Neurology, The First Affiliated Hospital of Shandong First Medical University & Shandong Provincial Qianfoshan Hospital, Jinan, Shandong, China

**Keywords:** cerebral hemodynamics, chronic large-vessel occlusion, collateral circulation, endovascular therapy, revascularization

Chronic large-vessel occlusion is best understood not as a static angiographic finding but as a dynamic cerebrovascular state in which anatomy, collateral adaptation, hemodynamic reserve, tissue vulnerability, and treatment strategy interact over time. This distinction matters clinically. Hemodynamic impairment has been associated with recurrent ischemic risk in carotid occlusive disease (1), yet randomized trials of extracranial-intracranial bypass, including COSS and CMOSS, have not demonstrated an overall benefit over medical therapy in selected patients with symptomatic carotid or middle cerebral artery occlusion (2, 3). Meanwhile, advances in endovascular recanalization have renewed interest in carefully selected patients with chronic occlusion, although the evidence remains largely observational (4). These apparently conflicting observations point to a central challenge: the presence of an occluded artery alone is insufficient to identify who will deteriorate, who may benefit from revascularization, and who may be harmed by intervention. Patients with similar angiographic lesions may have markedly different clinical trajectories depending on collateral sufficiency and residual perfusion. Accordingly, diagnosis should move beyond defining the site and length of occlusion toward characterizing its physiological consequences and the viability of the threatened vascular territory.

The contributions to this Research Topic, taken together, extend beyond chronic occlusion itself and support a more useful continuum-based framework for large-vessel occlusive disease—from vascular remodeling and collateral compensation, through patient selection and revascularization, to procedural execution and post-reperfusion recovery. Collateral biology is a logical starting point. Li et al. review postoperative collateral formation in moyamoya disease, a prototypical chronic progressive steno-occlusive arteriopathy, and highlight the interplay of genetic susceptibility, angiogenic signaling, cellular mechanisms, and clinical factors. Their synthesis reinforces an important concept: successful revascularization depends not only on restoring a conduit for blood flow, but also on the biological capacity of the cerebral circulation to adapt. This is consistent with contemporary views of moyamoya disease as a heterogeneous disorder in which mechanisms, hemodynamics, and response to revascularization vary substantially across patients (5). For chronic large-vessel occlusion more broadly, anatomical severity should therefore be interpreted together with collateral status, perfusion, and cerebrovascular reserve rather than in isolation.

A second theme is the transition from a binary question—whether to revascularize—to a more precise question: which patient, which lesion, and which strategy? Qureshi et al. explored a potentially higher-risk subgroup of patients with extracranial vertebral artery stenosis treated soon after ischemic stroke. The primary vertebrobasilar stroke/death endpoint did not reach conventional statistical significance, although exploratory outcomes favored stenting, appropriately framing the findings as hypothesis-generating rather than definitive. This nuance is important because pooled randomized evidence has continued to show uncertainty regarding the clinical benefit of vertebral artery stenting (6). Dong et al. approach the same problem from a procedural perspective, evaluating drug-coated balloon angioplasty for symptomatic vertebral artery origin stenosis and proposing a pragmatic DCB-first strategy with selective bailout stenting. Together, these studies exemplify a broader move toward lesion-specific and adaptive revascularization rather than routine device-based treatment.

Technical execution forms a third component of this framework. Liao et al. report balloon-assisted thrombectomy for acute middle cerebral artery occlusion caused by thrombus superimposed on intracranial atherosclerotic stenosis and identify shorter puncture-to-reperfusion time as an important correlate of favorable outcome. Sun et al. show that ultrasound guidance can facilitate transradial cerebral angiography by reducing difficult access and procedure time. Hu et al. in a rare tandem embolic stroke with free-floating thrombus, illustrate the value of comprehensive vascular imaging and a staged, multimodal endovascular strategy when pathology spans multiple vascular segments. These studies concern acute intervention rather than chronic occlusion *per se*, but they provide a transferable principle: once revascularization is considered appropriate, access planning, lesion morphology, procedural efficiency, and rescue strategy can influence whether technical success becomes meaningful tissue reperfusion.

The final theme is equally important: recanalization is not synonymous with recovery. Hu et al. identify an association between lower atherogenic index of plasma and hemorrhagic transformation after endovascular treatment of large-artery atherosclerotic stroke, suggesting that systemic biological factors may contribute to post-reperfusion vulnerability. Yuan et al. review prediction models for malignant cerebral edema after thrombectomy and show that commonly used clinical and imaging predictors coexist with substantial methodological bias and limited external validation. Yang et al. demonstrate that the 24-h NIHSS may provide stronger prognostic information than baseline or change scores after successful thrombectomy in cardioembolic anterior-circulation occlusion. Collectively, these studies shift attention from opening the artery to understanding the biological and neurological response after flow restoration. This distinction is particularly relevant to chronically hypoperfused tissue, in which abrupt changes in perfusion may expose vulnerabilities not captured by angiographic recanalization grades alone.

We therefore view the central message of this Research Topic as one of precision rather than procedural expansion. Future studies should standardize definitions of chronicity and technical success, quantify hemodynamic failure and collateral capacity, distinguish embolic from low-flow mechanisms, and prospectively validate multimodal models that integrate vascular anatomy, perfusion, clinical phenotype, and biological markers. Most importantly, chronic occlusion-specific evidence still requires prospective multicenter cohorts and randomized studies capable of identifying treatment-responsive subgroups. The goal is not simply to reopen an artery. It is to determine when restoring flow can be achieved safely, for the right mechanism and the right patient, and translated into durable neurological benefit.
